# Research and progress of microRNA-136 in metastatic tumors

**DOI:** 10.3389/fonc.2025.1555270

**Published:** 2025-03-04

**Authors:** Chenwen Wang, Zixiong Chen, Wei Ni, Jiang Wang, Wei Zhou

**Affiliations:** ^1^ Department of Orthopedics, Liyuan Hospital, Tongji Medical College, Huazhong University of Science and Technology, Wuhan, Hubei, China; ^2^ Affiliated Suzhou Hospital of Nanjing Medical University, Suzhou Municipal Hospital, Gusu School, Nanjing Medical University, Suzhou, China; ^3^ Department of Orthopedics, Tongji Hospital, Tongji Medical College, Huazhong University of Science and Technology, Wuhan, Hubei, China

**Keywords:** miRNA, miR-136, metastatic tumors, target, immunotherapy

## Abstract

**Background:**

MiR-136 is abnormally expressed in many types of metastatic tumors and is closely associated with tumor cell proliferation, apoptosis, invasion, and metastasis, indicating its important role in tumor development and progression. This review summarizes current knowledge regarding miR-136’s molecular mechanisms, functional roles, and impact on chemotherapy in different human cancers.

**Methods:**

A literature search was conducted in PubMed and Web of Science using “miR-136” and “metastatic tumors” as English keywords, and in CNKI and Wanfang databases using the same terms in Chinese. Studies related to miR-136 research in metastatic tumors and high-quality evidence from similar studies were included. Meta-analyses, dissertations, conference papers, low-quality articles, unavailable full-text articles, and republished articles were excluded.

**Results:**

This review synthesizes the current understanding of miR-136’s role in various cancers, including osteosarcoma, gastric cancer, gallbladder cancer, esophageal cancer, prostate cancer, colorectal cancer, breast cancer, glioma, and thyroid cancer. miR-136 acts as a tumor suppressor by targeting various genes, including MTDH, PTEN, MAP2K4, MUC1, LRH-1, MIEN1, RASAL2, CYR61, and KLF7. It influences multiple signaling pathways, including the ERK/mitogen-activated protein kinase, Wnt/β-catenin, Ha-Ras, PI3K/Akt, Aurora-A kinase, nuclear factor-κB, and JNK pathways. Furthermore, miR-136 is involved in chemoresistance by modulating ROCK1, PPP2R2A, and the miR-136-Notch3 signaling axis.

**Conclusions:**

MiR-136 demonstrates promising potential as a novel biomarker and therapeutic target in various human cancers. Further research is needed to fully elucidate its complex roles in cancer development, progression, and drug resistance, particularly regarding its potential in immunotherapy.

## 1 Introduction

MicroRNAs (miRNAs) are a class of non-coding, single-stranded RNA molecules, approximately 22 nucleotides long, encoded by endogenous genes. They participate in the transcriptional regulation of gene expression in animals and plants (1). By recognizing and binding to the 3′ untranslated region of target gene mRNA, miRNAs inhibit mRNA translation or promote degradation (2, 3). miRNAs can also affect histone modifications and promoter site methylation to regulate target gene expression (4, 5). A single miRNA can regulate the expression of multiple target genes (6, 7), by modulating various mRNAs, miRNAs functionally participate in a range of physiological and pathological processes, including cell differentiation, proliferation, migration, and apoptosis (8, 9). Abnormal miRNA expression has been reported in various types of metastatic tumors (10, 11), suggesting their potential oncogenic or tumor-suppressive functions. MiR-136 is abnormally expressed in many types of metastatic tumors and is closely associated with tumor cell proliferation, apoptosis, invasion, and metastasis, indicating its significant role in tumor development and progression (12–21). Moreover, miR-136 contributes to cancer resistance to various chemotherapeutic agents (21–24). Given its critical involvement in tumor biology, miR-136 represents a promising biomarker and therapeutic target for early cancer detection and treatment. This review summarizes the signaling pathways and mechanisms of action of miR-136 in various metastatic tumors, providing a comprehensive understanding of its role in metastatic tumors.

## 2 Literature search strategy

**Figure 1:**
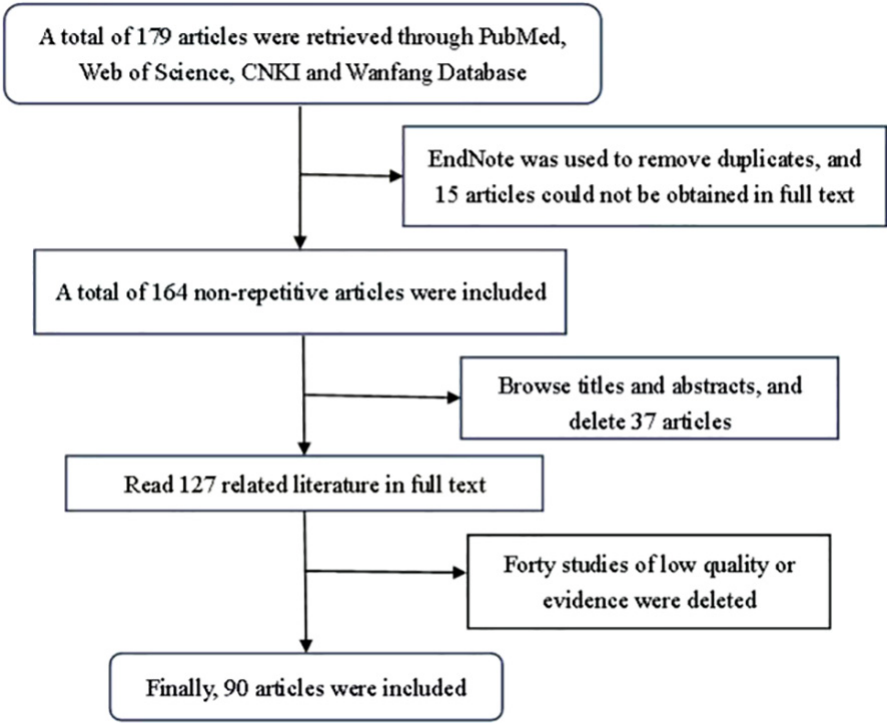
Literature search strategy.

This review was indexed in PubMed and Web of Science using “miR-136” and “metastatic tumors” as English keywords. “miR-136” and “metastatic tumors” were used as Chinese keywords in CNKI and Wanfang databases. Studies were included based on the following criteria: (1) relevance to miR-136 research in metastatic tumors; and (2) provision of high-quality evidence. The following exclusion criteria were applied: (1) studies not published in English or Chinese; (2) meta-analysis, dissertations, conference papers, and (3) low-quality, unavailable full-text and republished articles. Studies were selected according to these criteria (**Figure 1**). miR-136 and the target genes in tumors are listed in **Table 1**.

**Table 1:** miR-136 and the target genes in tumors.

Cancers/tumors	Target genes
osteosarcoma	MTDH
gastric cancer	PTEN
gallbladder cancer	MAP2K4
esophageal cancer	MUC1
prostate cancer	MAP2K4
colon cancer	LRH-1
	MIEN1
Triple-negative breast cancer	RASAL2
glioma	CYR61
	KLF7
thyroid cancer	MTDH

## 3 Role of miR-136-5p in cancers

### 3.1 miR-136 and metastatic osteosarcoma

Metastatic osteosarcoma (OS) is the third most common primary bone malignancy in children and adolescents (25), and commonly occurs in the proximal tibia, humerus, and metaphyseal region of the distal femur (26). The MTDH gene, located on human chromosome 8, facilitates tumor cells adhesion to distant blood vessels, playing a critical role in cancer spread and metastasis. MTDH is a involved in several oncogenic signaling pathways, such as ERK/mitogen-activated protein kinase, Wnt/β-catenin pathway, Ha-Ras and PI3K/Akt pathways, Aurora-A kinase signaling pathway, and nuclear factor-κB signaling pathway (27, 28). miR-136 inhibits the proliferation, invasion, and migration of osteosarcoma cells by negatively regulating MTDH.

### 3.2 miR-136 and metastatic gastric cancer

Metastatic gastric cancer (GC) has the highest mortality rate among patients in China (29). Various pathogenic factors contribute to GC development and progression, including environmental factors, diet, infection, and genetic mutations, particularly the abnormal expression of proto-oncogenes or tumor suppressor genes (30). Compared with adjacent non-neoplastic gastric mucosal tissues and normal gastric epithelial cells, miR-136 expression was significantly increased while PTEN mRNA expression was decreased (19). Bioinformatics analysis has identified PTEN as a target of miR-136. Following miR-136 knockdown, PTEN mRNA and protein expression levels increase, whereas p-AKT protein levels decrease. PTEN expression was negatively correlated with miR-136 expression. PTEN negatively regulates various biological processes through the PI3K/AKT signaling pathway, including cell proliferation, migration, invasion, and apoptosis (31–34). Therefore, inhibiting miR-136 can suppress the proliferation, invasion, and metastasis of gastric cancer cells by modulating the PTEN/AKT/p-AKT signaling pathway.

### 3.3 miR-136 and metastatic gallbladder cancer

Metastatic gallbladder cancer (GBC) is the most common malignancy of the biliary tract and the fifth most common tumor of the digestive tract (35). Compared with normal gallbladder epithelial cells, miR-136 is expressed at lower levels in gallbladder cancer cells (36). Mitogen-activated protein kinase 4 (MAP2K4) has been identified as a target gene of miR-136, which activates the JNK signaling pathway, a key mediator in tumorigenesis and apoptosis (36–38). In gallbladder cancer, miR-136 overexpression inhibits the MAP2K4-mediated JNK signaling pathway, thereby regulating the expression of downstream genes (39). Inhibition of the JNK signaling pathway decreases vascular endothelial growth factor (VEGF) expression, negatively regulating tumor growth and metastasis, as VEGF activates the angiogenesis signaling cascade and promotes tumor vascular endothelial cell proliferation, differentiation, and migration (40). Simultaneous inhibition of the JNK signaling pathway increases the ratio of c-caspase-3/t-caspase-3 and c-caspase-9/t-caspase-9, thereby promoting apoptosis (41, 42). Therefore, miR-136 overexpression inhibits angiogenesis and cell proliferation in gallbladder cancer while promoting apoptosis, suggesting a potential therapeutic role in gallbladder cancer treatment.

### 3.4 miR-136 and metastatic esophageal cancer

Metastatic esophageal cancer (EC) is the eighth most common cancer and the sixth leading cause of cancer-related deaths worldwide (43), EC comprises two main subtypes: esophageal adenocarcinoma (EAC), which has an increasing incidence, and esophageal squamous cell carcinoma (ESCC), prevalent in East Africa, Central Asia, and China (44). miR-136 expression is reduced in ESCC tissues, while MUC1 mRNA and protein expression levels are elevated compared to those in adjacent normal tissues. Bioinformatic analysis and luciferase activity assays confirm MUC1 as a miR-136 target, with an inverse correlation between their expression levels (45). miRNAs bind to the 3′ untranslated region (3′-UTR) of their target mRNAs, reducing their stability and post-transcriptional expression, thereby influencing biological processes such as cell growth, proliferation, differentiation, and death (46). Additionally, miR-136 upregulation reduces survival, inhibits colony formation, and induces apoptosis in ESCC cells under irradiation, whereas MUC1 upregulation reverses these effects. miRNAs can also influence cellular responses to precision drugs by interfering with DNA repair and drug targets (47).

### 3.5 miR-136 and metastatic prostate cancer

Metastatic prostate cancer (PCa) is a common malignancy and the second most prevalent tumor of the urinary and reproductive systems (48). The occurrence and progression of PCa are regulated by miRNAs, and PCa-related miRNAs research provides novel biomarkers for diagnosis and treatment (49, 50). miR-136 expression is reduced in PCa tissues and cell lines, whereas its upregulation inhibits PCa cells. A luciferase reporter assay has confirmed that mitogen-activated protein kinase 4 (MAP2K4) is a miR-136 target gene. MAP2K4 is upregulated in PCa tissues, and its expression levels are inversely correlated with miR-136 levels. MAP2K4, located on chromosome 17, is involved in various tumorigenic and pathophysiological processes, including cell proliferation, invasion, metastasis, and apoptosis (51–53). In PCa, MAP2K4 overexpression promotes cell proliferation and metastasis while inhibiting G1-S phase arrest and apoptosis (39, 54).Thus, miR-136 may suppress PCa proliferation and invasion by targeting MAP2K4, making it a potential candidate for PCa therapy.

### 3.6 miR-136 and metastatic colorectal cancer

Metastatic colon cancer (CC) is one of the most prevalent malignancies and the fourth leading cause of cancer-related deaths worldwide (55). Hepatic receptor homolog-1 (LRH-1), a member of the nuclear receptor subfamily, is a recognized oncogene in many cancers (56), promoting the proliferation, invasion, and migration of cancer cells (57). LRH-1 plays a crucial role in various biological processes, including bile acid homeostasis, reverse cholesterol transport, steroid production, differentiation, and development (58). LRH-1 knockdown has been shown to inhibit colon cancer cell proliferation and induce G0/G1 cell cycle arrest (59). Additionally, LRH-1 promotes colon cancer cell growth by inhibiting the recruitment of p53 to the promoter of the cell cycle inhibitor p21 (60). Wnt signaling is aberrantly activated in approximately 80% of colon cancers (61), and its downstream genes, including cyclin D1, cyclin E1, and c-Myc, are implicated in the proliferation and metastasis of colon cancer cells (62). LRH-1 is a novel co-activator of Wnt signaling pathway transduction (59, 63), and can interact with transcription factor 4 and β-catenin to promote the expression of cyclin D1, cyclin E1, and c-Myc (63). miR-136 offers a novel pathway for the inhibition of Wnt signaling by significantly reducing the expression of cyclin D1, cyclin E1, and c-Myc in colon cancer through LRH-1 suppression (20).

MIEN1, located in the 17q12 region of the chromosome near the Her-2/neu locus (64), is frequently dysregulated in various cancers (65, 66). MIEN1 expression is elevated in colorectal cancer tissues and is closely associated with tumor serous invasion, lymph node metastasis, and advanced Dukes stage (67). miR-136 has been shown to inhibit colon cancer cell invasion, migration, and EMT progression by regulating the Akt/NF-κB signaling pathway through its target gene, MIEN1 (66).

### 3.7 miR-136 and metastatic breast cancer

Metastatic breast cancer (BC) is the most common cancer in women and a leading cause of cancer-related deaths (68, 69). Triple-negative breast cancer (TNBC) is a heterogeneous group of breast cancers characterized by the loss of estrogen receptor (ER), progesterone receptor (PR), and human epidermal growth factor receptor 2 (HER2) gene expression (70). The Ras pathway is one of the most commonly dysregulated pathways in cancer, with Ras protein mutations occurring at high frequency (71). Ras activity is negatively regulated by Ras GTPase-a activating proteins (RasGAPs), which catalyze the hydrolysis of Ras-GTP to Ras-GDP (72). Interestingly, RASAL2, a GAP, has been identified as an oncogene promoting tumor production and metastasis in various cancers (73–75), However, rather than suppressing tumors, RASAL2 facilitates mesenchymal invasion and metastasis (73, 76, 77). In TNBC, miR-136 has been shown to act as a tumor suppressor by directly targeting RASAL2. Through downregulation of RASAL2, miR-136 effectively inhibits tumor growth and metastasis, underscoring its therapeutic potential in TNBC.

### 3.8 miR-136 and metastatic glioma

Metastatic glioma is a common malignancy (78, 79). Bioinformatic studies suggest that miR-136 can function as either a tumor suppressor or an oncomiR, depending on the context. Overexpression of miR-136 has been shown to inhibit glioblastoma cell proliferation by targeting CYR61. Signal transduction via the mTOR pathway is activated alongside miR-136 expression and is dependent on the activities of AKT, ERK1/2, and mTORC1. miR-136 expression is reduced in glioma tissues compared to adjacent normal tissues. KLF7, a target gene of miR-136 (18), promotes polyamine biosynthesis and glioma progression by activating arginine succinate lyase (80). Overexpression of miR-136 has been shown to inhibit glioma cell growth and migration.

### 3.9 miR-136 and metastatic thyroid cancer

Metastatic thyroid cancer (TC) is a common endocrine neoplasm, accounting for approximately 3.1% of all human malignancies (81). Papillary thyroid cancer, the most prevalent pathological subtype of TC, constitutes approximately 80% of cases. Its incidence is higher than that of other subtypes, such as anaplastic, follicular, and medullary thyroid cancer. The incidence of TC has increased in many countries. Although the overall mortality rate of TC is relatively low, with a 5-year survival rate of 98%, the clinical outcomes of advanced TC remain poor. Nearly half of patients with distant TC metastases die within 5 years of diagnosis. Recurrence and lung metastasis remain the leading causes of mortality in TC patients. Studies have reported higher levels of miR-136 in papillary adenocarcinomas compared to benign nodular goiter (82). MTDH has been identified as a target gene in various cancers (13, 17), with its overexpression playing a key role in cancer development. A luciferase reporter assay has confirmed the targeted regulatory relationship between miR-136-5p and MTDH (15).

## 4 miR-136 and chemoresistance in tumors

Chemotherapy is one of the most common clinical treatments for tumors; however, chemoresistance remains a widespread challenge (83, 84). Cisplatin, one of the most effective chemotherapeutic agents (85), inevitably encounters drug resistance, limiting the efficacy of other agents and leading to potential treatment failure. Abnormal miRNA expression can disrupt the regulation of chemotherapy drug target proteins, ultimately contributing to drug resistance. miR-136, one of the most extensively studied miRNAs, is abnormally expressed in various tumors. miR-136 overexpression reduces ROCK1 expression in cisplatin-treated tumor cells and attenuates the Akt/mTOR signaling pathway, leading to chemoresistance (86). Additionally, miR-136 overexpression promotes anlotinib resistance in non-small cell lung cancer by targeting PPP2R2A, thereby activating the Akt pathway. miR-136 can be transferred from anlotinib-resistant cells to anlotinib-sensitive cells via exosomes, inducing drug resistance and promoting cell proliferation (22). In gliocytomas, miR-136 overexpression enhances temozolomide cytotoxicity (24). In ovarian cancer, the miR-136-Notch3 signaling axis plays a crucial role in the development of chemoresistance (87). These findings suggest that miRNA-mediated chemoresistance and chemo sensitization can be modulated to enhance chemotherapy efficacy, offering new strategies for overcoming tumor drug resistance.

## 5 miR-136 and immunotherapy

Immunotherapy is one of the most effective cancer treatment strategies. Among immune checkpoint molecules, miRNA-based PD-L1 regulation is the most widely studied. PD-L1, expressed on immune and cancer cell surfaces, inhibits T-cell proliferation by binding to its receptor, PD-1. In NSCLC, miR-34a directly binds to the 3’-UTR of PD-L1, inhibiting its expression. miR-140 functions as a PD-L1 modulator in osteosarcoma (88), whereas miR-15a and miR-15b exert antitumor effects by blocking PD-L1 in neuroblastoma (89). In breast cancer cells, PD-L1 activates PDCD4 via the PI3K/Akt pathway, a process significantly enhanced by miR-21 (90). However, the role of miR-136 in tumor immunotherapy remains unclear and requires further investigation.

## 6 miR-136 and other miRNAs

miR-136 stands out for its involvement in epigenetic regulation (e.g., targeting EZH2) and its ability to enhance chemosensitivity in cancer cells. Like miR-34a and let-7, miR-136 acts as a tumor suppressor by targeting anti-apoptotic proteins and inhibiting proliferation. Similar to miR-21 and miR-155, miR-136 regulates the PI3K/AKT pathway, but it does so in a tumor-suppressive manner, unlike the pro-tumorigenic effects of miR-21 and miR-155. In conclusion, miR-136 shares some functional roles with other miRNAs in cancer, such as regulating apoptosis and proliferation, but its unique involvement in epigenetic regulation and chemosensitivity distinguishes it from other miRNAs. Its downregulation in cancer highlights its potential as a therapeutic target or biomarker.

## 7 Summary

MiRNAs regulate the expression of their corresponding target genes and exhibit direct or indirect carcinogenic or tumor-suppressive effects in various cancers. Many studies have identified miR-136 as a tumor suppressor gene. miR-136 is an anti-invasive miRNA that inhibits mesenchymal invasion and transfer in TNBC. The miR-136/RASAL2/MET axis functions as a repressor of TNBC metastasis. miR-136 also negatively regulates colon cancer progression by targeting LRH-1, preventing aberrant activation of Wnt signaling. In gliomas, miR-136 inhibits proliferation and induces apoptosis by regulating AEG-1 and BCL-2 gene expression. Inhibition of miR-136 expression upregulates its target AEG-1 gene and significantly improves the metastatic ability of hepatoma cells. Additionally, miR-136 suppresses lung cancer cell proliferation, invasion, and migration. In osteosarcoma, it negatively regulates MTDH, exerting tumor-suppressive effects. miR-136 also inhibits the MAP2K4-mediated JNK signaling pathway, thereby influencing downstream gene expression. These findings suggest that miR-136 has broad tumor-suppressive roles, making it a potential therapeutic target. Furthermore, miRNAs regulate multiple target genes, underscoring their functional diversity and importance in cancer treatment.

## 8 Future outlook

miR-136 plays a role in various signaling pathways in cancer and has been identified as a promising biomarker for cancer diagnosis and prognosis. Additionally, several cell-based and preclinical studies have shown that blocking or inhibiting miR-136 can lead to the regression of various cancer types, making it a strong candidate for cancer drug discovery. miR-136 is also actively involved in regulating drug resistance, and any effective miR-136 targeting strategy could help reduce cancer cell resistance and recurrence. Several small-molecule inhibitors of miR-136 have been reported; however, its functions in both cancerous and normal cells are not fully understood. Using advanced sequencing techniques and powerful bioinformatics tools to explore the regulatory function of miR-136 in complex oncogenic pathways will enhance our understanding and reveal new potential applications for miR-136 in cancer diagnosis and treatment. Beyond investigating miR-136 itself, exploring effective drug delivery methods is also essential. The emerging research focus on exosomes offers new avenues for drug delivery. Exosomes are messengers in cell-to-cell communication in the tumor microenvironment, and exosomal circRNAs have been reported to function as miRNA sponges, which are important in tumors. However, the role of miR-136 in human cancer requires further investigation. This review summarizes miR-136’s feasibility as a diagnostic or prognostic biomarker and provides new perspectives on cancer resistance and drug susceptibility research.
